# Actual Politics on Physical Activity Challenged by Crisis. The Italian Case of Reaction to the COVID-19 Pandemic

**DOI:** 10.3389/fsoc.2020.566885

**Published:** 2020-12-15

**Authors:** Federico Ranieri

**Affiliations:** Department of Neuroscience, Biomedicine and Movement Sciences, University of Verona, Verona, Italy

**Keywords:** promotion of physical activity, stigma, movement restriction, social distancing, SARS-CoV-2, noncommunicable diseases

## Introduction

In 2018, the World Health Organization (WHO) promoted a global action plan on physical activity as a primary health preservation strategy and estimated a 280% total return on investments to obtain a 15% relative reduction in the global prevalence of physical inactivity by 2030 (World Health Organization, 2018a,b). Indeed, physical inactivity is estimated to account for 1–3% of national health-care costs, excluding those associated with mental and musculoskeletal conditions (World Health Organization, 2018a). The potential economic return derives from multiple factors, including prevention and treatment of noncommunicable diseases, improved mental health, indirect benefits from reduced pollution, increased earning capacity, increased public resources, and reduced health-care expenditure (World Health Organization, 2018b). To these ends, the WHO recommends “*a ‘systems-based' approach with a strategic combination of ‘upstream' policy actions aimed at improving the social, cultural, economic and environmental factors that support physical activity, combined with ‘downstream,' individually focused (educational and informational) approaches*.” (World Health Organization, 2018a).

In March 2020, the outbreak of the SARS-CoV-2 pandemic forced most countries all over the world to limit freedom of movement and outdoor activities. Here, I analyze the possible outlasting impact of emergency interventions on the psychosocial dynamics governing promotion of physical activity. The Italian case is taken as representative of dynamics that can replicate in different countries over the world.

After the initial outbreak in China, Italy was the first European country to face a rapid increase of infections, and it reacted with strict social distancing measures (Boccia et al., 2020). Starting from March 9, 2020, the entire country was put under lockdown, and going out was allowed only for strict necessities. While national legislation preserved the possibility of performing physical activity individually and in the proximity of the house, within the scope of “daily necessities,” many local administrations invoked the state of necessity to prohibit any kind of outdoor activity such as walking or running. The respect of rules was ensured with police rounds and drones and with a diffuse campaign of “stay home” publicity. Therefore, millions of people were forced to a reduced level of aerobic exercise. Starting from the first half of April, restrictions on physical activity were finally mitigated toward a return to normality.

## Impact of Emergency Response on Physical Activity

In the WHO recommendations (World Health Organization, 2018a), facilitating physical activity is based on four strategic objectives: (1) “active societies” through communication campaigns, mass participation initiatives, and training of involved professionals; (2) “active environments” for equitable access of all people to safe spaces; (3) “active people” by creating opportunities for regular physical activity; and (4) “active systems” through governance, resource mobilization, and coordinated actions.

In Italy, the importance of physical activity has been recognized since the 2003–2005 National Health Program, and it was confirmed in the following programs and in the National Prevention Plans, inspired to the WHO European Office policies. These plans acknowledge the relationship between a sedentary lifestyle and worsening of noncommunicable disorders (De Mei et al., 2018). Based on economic analyses (Centre for Economics Business Research, 2015), physical inactivity accounts for 14.6% of deaths in Italy and for a direct cost for the National Health Service of about 1.6 billion euros per year (by considering the four major diseases influenced by physical inactivity: breast and colon cancer, type 2 diabetes, and coronary artery disease). Total cost, including loss of productivity, is estimated to be much higher, in the order of 32 billion euros, corresponding to about 2% of gross national product (De Mei et al., 2018). In a latest report on the sports practice in Italy by the National Statistics Institute, it emerges that, in 2015, ~39% of the population is totally inactive, ~26% practices only some kind of physical activity, and ~35% practices sports occasionally or continuously. Data of previous years indicate that from 1995 to 2015, the number of people practicing sports increased to the expense of those performing only some kind of activity, while the number of inactive people was substantially unchanged (Italian National Statistics Institute, 2015).

The implementation of different lines of interventions is within the competence of the Ministry of Health, the Government Sport Office, and the Italian National Olympic Committee, a public noneconomic institution supervised by the head of government. The National Institutes of Health performs control, research, and counseling activities for the Ministry of Health. Moreover, the recently created “Sports and Health” public company, owned by the Ministry of Economic Affairs, is intended to translate into action public health policies related to physical activity. Among interventions in the last 2 years, particular attention has been paid to the promotion of physical activity among disadvantaged population, especially through financing of facilities in suburban areas or small towns. Other usual activities include financing of nonprofessional sport associations and organization and promotion of big sport events, such as the Olympic games and the European championships.

When facing current crisis, the “stay home” message recurred as a key element in the strategic narrative in Italy and in most other countries, being stressed as the necessary behavior to succeed in the so-called “war” against the pandemic and legitimating exceptional restrictions and penalties. In this context, the decision of banning outdoor activities, even performed individually and outside interdicted areas, was essentially taken to prevent violation of social distancing measures (i.e., by people invoking physical activity as a justification for staying out and meeting others). Thus, the restriction on physical activity appears as a fear-driven decision to ensure absolute control, facing the risk of breakdown of intensive care units (<1.0 bed available every 10,000 inhabitants). Indeed, a direct effect on limiting infections is unlikely, considering that virus transmission through droplets is prominent at short distance and in confined environments (World Health Organization, 2020). To confirm this assumption, the Italian National Institute of Health reports that most traced contagions diagnosed after lockdown mitigation continued to happen inside closed communities (May 1–20: 49.5% nursing homes; 24.3% private houses; 7.2% hospitals—June 1–30: 35.1% nursing homes; 24.6% private houses/relatives; 6.6% hospitals) (Italian National Institute of Health (ISS), 2020a,b).

While the pandemic can be resisted effectively by ensuring observation of social distancing rules and contact tracing (Giordano et al., 2020), there is risk of a negative balance to pay for disproportionate restrictions of movement in terms of efficacy of plans on physical activity, due to several reasons.

The first direct impact is on the “active people” objective, with consequent threats on physical and mental health. Despite several initiatives promoting home training, the overall reduction of aerobic exercise is destined to increase the burden of many chronic diseases. This concerns not only cardiovascular, metabolic, immunological, and musculoskeletal disorders but also brain health, with an expected impact on cognitive function throughout a person's lifespan and on people affected by cognitive decline (Hillman et al., 2008; Liu et al., 2019). Moreover, forced and prolonged home confinement, and restriction of individual liberty, can cause mental distress, especially for people already at risk (Gallagher, 2014; Alonzi et al., 2020; Brooks et al., 2020).

Second, the stigma with sports might derive from the implicit misleading message that physical activity must unavoidably be sacrificed to protect human life. Hence, physically active people are marked as unacceptably different and devalued (Goffman, 1963). Indeed, movement restrictions have been frequently associated with narrative by mass media and by some institutional representatives pointing to physically active people as the negative example of those neglecting the “stay home” directive, hence as a danger for the community, or depicting them as persons unrespectful of others' sufferance. This kind of narrative is believed to have found a fertile ground in the high prevalence of physical inactivity (Guthold et al., 2018) and poor health literacy among population (Paakkari and Okan, 2020), and to have facilitated the diffusion of intolerance sentiments leading to hate speech and cases of verbal and physical aggressions, as reported by media. This creates a condition in which fear for an invisible enemy (the virus) easily turns into hate for a physical enemy, for example, the runner, the perfect scapegoat for the pandemic. The risk here is amplifying social tensions and reducing the value attributed to active lifestyle (the “active societies” objective) well beyond the emergency.

Third, it should also be considered whether it is ethically acceptable to limit the rights of a large number of people because someone will not respect rules. Yet, unjustified restrictions of individual liberty might have even worse general consequences on the value attributed to human rights, creating a base for inequalities. The issue has even been raised that in fragile democracies, there is a risk that exceptional situations undermine human rights in the long-term (Nay, 2020). It must be clarified that the loss of value of human rights is usually far from the aims of governments' decisions. This is exemplified in one Italian Prime Minister's speech to parliament, on April 30, 2020, in which it is stressed that all exceptional and urgent decisions were carefully taken to protect human life, based on available scientific data (Italian Prime Minister's Speech to Parliament on the Restart of Economic Activities, 2020). It may then happen, through the communication chain, that the valuable and easily agreeable assertion that human life comes first, when put out of its specific context and taken as an absolute reference, favors the acceptance by the public opinion that all other rights can be sacrificed, without critically evaluating if they really threaten human life.

**Figure 1:**
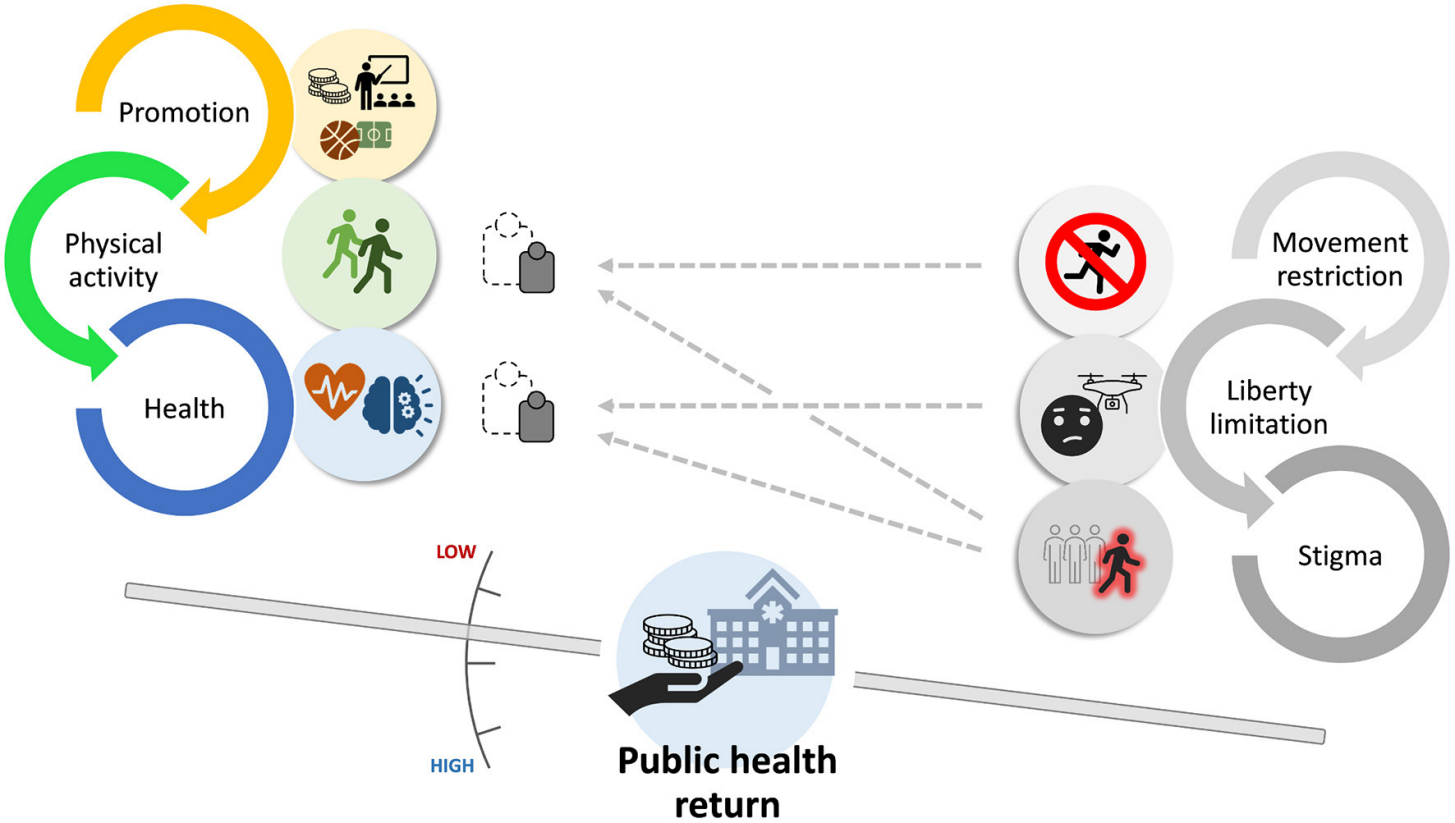
Dynamics of health promotion through physical activity influenced by emergency restrictions. Promotion (through funding, communication, environment, facilities, and governance) leads to increased number of physically active people and to better physical and mental health, and hence to monetizable returns for the public health system. Movement restriction, limitation of individual liberty, and stigma to active people directly or indirectly reduce the weight of physical activity and health outcome, thus depreciating promotion efforts and lowering long-term public health return. Stronger plans on physical activity will be more resilient to disqualifying influences.

All the above analyzed conditions can strongly depreciate the efforts toward active life promotion and, in turn, the occurrence of these conditions can be facilitated by vulnerabilities in politics on physical activity (Figure 1).

## Conclusions

Crises typically expose vulnerabilities. In Italy, the pandemic emergency uncovered risks of failure of long-term plans on physical activity, making manifest that they are not valued as part of the upstream of health protection strategy. While the general directives strongly point to promotion of physical activity, actual policies are dramatically challenged. This weakness, while revealed by crisis, remains as a predictor of failure independently from current emergency. It is assumed that the phenomena analyzed here characterize a dynamic that can be found elsewhere in the world.

This analysis does not put on balance health protection from the pandemic on one side (by means of social distancing) and from physical inactivity on the other; rather, it concludes that the control of the pandemic can be achieved while preserving physical activity in compliance with social distancing rules. Indeed, the consequences of disproportionate restrictions and of negative narratives risk to generate a public health damage that go beyond the reduced level of activity during lockdown.

The main threat to politics on physical activity appears to be in the intrinsic incoherence of promotion strategies, where physical activity is communicated as a main objective, but it is actually undervalued. This creates a vicious circle with exogenous factors, such as any kind of barriers and stigma, further reducing the power of promotion strategies. Disparity between national and regional legislations also emerges as a critical factor for the efficacy of policy actions.

By revealing weaknesses, a crisis turns into an occasion for future improvement. For an effective promotion of physical activity, the following are proposed as key elements to preserve:

1. Keeping a long-time vision. Interventions should have a decade perspective: returns should be evaluated in the long-term perspective and not on a day-by-day basis; when facing dramatic contingencies, policies should be adapted to the extent that is strictly necessary.
2. Keeping a coherent action, ensuring that local measures do not contrast with national measures or with general principles, unless it cannot be avoided due to local contingencies.
3. Keeping a coherent communication, by avoiding delivering negative or incoherent messages affecting the perception of the value of physical activity among society.
4. Promoting active interventions to counteract an infodemic of misinformation.

## Author Contributions

FR entirely conceived and prepared the manuscript.

## Conflict of Interest

The author declares that the research was conducted in the absence of any commercial or financial relationships that could be construed as a potential conflict of interest.
